# *“Small small interventions, big big roles”*- a qualitative study of patient, care-giver and health-care worker experiences of a palliative care programme in Kerala, India

**DOI:** 10.1186/s12904-019-0400-2

**Published:** 2019-02-04

**Authors:** Rekha Rachel Philip, Emilie Venables, Abdulla Manima, Jaya Prasad Tripathy, Sairu Philip

**Affiliations:** 1 Department of Community Medicine, Government T.D Medical College Alappuzha, Vandanam P.O, Alappuzha, Kerala India; 2 Médecins Sans Frontières - Operational Centre Brussels, Medical Department, Operational Research Unit (LuxOR), Luxembourg, Luxembourg; 3 Division of Social and Behavioural Sciences, School of Public Health and Family Medicine, University of Cape Town, Cape Town, South Africa; 4 Malappuram Initiative in Palliative care, Malappuram, Kerala India; 5 International Union Against Tuberculosis and Lung Disease, Paris, France; 6 Department of Community Medicine, All India Institute of Medical Sciences, Nagpur, India

**Keywords:** Palliative care, Home-based care, Qualitative research, Care-givers, India

## Abstract

**Background:**

Home-based palliative care is an essential resource for many communities. We conducted a qualitative study to explore perceptions of a home-based palliative care programme in Kerala, India, from the perspective of patients, their care-givers and the doctors, nurses and volunteers running the intervention.

**Methods:**

A descriptive qualitative study was carried out. One focus group discussion (FGD) was conducted with patients (*n* = 8) and two with male and female volunteers (*n* = 12); and interviews were conducted with doctors (*n* = 3), nurses (n = 3) and care-givers (*n* = 14). FGDs and interviews were conducted in Malayalam, audio-recorded, transcribed verbatim and translated into English. Transcripts were coded and analysed using manual content analysis.

**Results:**

Doctors, nurses and volunteers have interdependent roles in providing palliative care to patients, including mentorship, training, patient care and advocating for patient needs. Volunteers also considered themselves to be mediators between families and the programme. Care-givers were mainly female and were caring for relatives. They have physically demanding, psychologically stressful and socially restrictive experiences of care-giving. They felt that the programme facilitated their role as care-givers by giving them training and support. Patients with long standing illnesses felt that the programme enabled them to become more independent and self-reliant. The local community supports the programme through economic contributions and offering practical assistance to patients.

**Conclusion:**

The salient features of this programme include the provision of regular holistic care through a team of doctors, nurses and patients. The programme was perceived to have improved the lives of patients and their care-givers. The involvement of volunteers from the local community was perceived as a strength of the programme, whilst simultaneously being a challenge.

## Background

In the context of ageing populations with growing palliative care needs, home-based care has become a policy priority in many countries [1]. Studies have shown that home-based palliative care services facilitate the preference to die at home while decreasing symptom burden [2–5]. Palliative care has also been reported to be beneficial in stabilising or slowing a number of long standing illnesses including neurodegenerative illness and chronic respiratory disorders [6, 7]. However, the notion that palliative care is only for those who are dying prevents many from accessing care [8].

A systematic review that evaluated the mechanism of home-based palliative care interventions has shown that these services facilitate feelings of normality and security at home through individualised care adapted to the family. However, it also stresses the need to appropriately design, deliver and report home palliative care interventions [9].

There are considerable differences between the models and provision of home-based palliative care interventions in different settings. Home-based palliative care usually provides holistic care through a multidisciplinary team consisting of physicians, nurses, personal support workers and case managers [10, 11]. Many of these services exist in high income countries with a huge gap in provision in low and middle-income countries. For example, in the South Asian Association of Regional Cooperation countries, which is home to one quarter of world’s population, palliative care services are documented only from three out of eight countries, namely India, Pakistan and Nepal. The majority of these services are from Kerala, India [12].

Home-based palliative care services are gaining popularity with care being taken to the doorstep of the patient. It is well suited to conditions in India where a family member is usually the primary care-giver of the sick person. In this socio-cultural context, the Kerala model of community-based palliative care has been described as an exemplar model for resource poor settings [13]. Since 1997, the State of Kerala has pioneered free of charge home-based palliative care through a socially innovative approach called the Neighbourhood Network in Palliative Care. Volunteers from the local community play a key role in the programme. They are trained to identify the psycho-social problems of people with chronic debilitating conditions and to intervene effectively with active support from a network of trained professionals in their area. The programme was first piloted with cancer patients in Malappuram, a northern district in Kerala. It was later expanded to include other conditions requiring long-term care such as cerebro-vascular accidents, dementia, paraplegia and psychiatric illness, rather than only providing end of life care, as in other palliative care programmes in other contexts [14–16]. The model was shown to be successful, and inspired the state to implement a palliative care policy to ensure universal coverage of palliative care services in all its local administrative units, making it the first state in Asia to develop such a policy [17].

Although this Kerala model of community-based palliative care has been well-described, the programme has not yet been evaluated to identify and share successful strategies and lessons learned [12]. Evaluations of successful care models such as the one in Kerala, India are worthwhile to identify the strengths and challenges of providing home based palliative care [9, 12].

We conducted a qualitative study to describe the experiences of patients, care-givers, programme staff and volunteers in providing and receiving care in one of the oldest community based palliative care units in India. We also explore the perceived benefits and challenges of the programme and the role of the community in implementation. This study was part of a larger evaluation of the palliative care services offered in this palliative care unit, and the quantitative component has been published elsewhere [16].

## Methods

### Study design

We used a descriptive qualitative study design involving in-depth interviews (IDIs) and Focus Group Discussions (FGDs) with patients, care-givers and health-care workers involved in the implementation of the programme. Qualitative descriptive design allows a straightforward description of study participant’s experiences in their own language, without the interpretation of existing theories [18, 19].

### Setting

Kerala is the southernmost state in India, with a population of around 330 million people. The state has the highest human development index (0.7); highest literacy rate (93%) and highest life expectancy (77 years) in the country [20, 21]. Community-based palliative care units exist in all districts in Kerala.

This study took place in the district of Malappuram in Kerala, where India’s first community based palliative care services were implemented. There are more than 100 palliative care units in the district, which are run by Community Based Organisations. Our study was conducted in the oldest palliative care unit of the district, the Manjeri Pain and Palliative Care Unit, which has been providing home-based services to patients with a range of long-term illnesses since 1997. There are currently 508 patients enrolled in the programme, who are cared for by a team of doctors, nurses and volunteers. The clinic provides free of charge home-based care to patients with terminal illnesses such as cancer, as well as those with longstanding conditions including paraplegia, cerebral palsy, psychiatric illness, stroke and the aged [16]. The clinic also assists patients who are marginalized from routine health care, such as those with psychiatric illness. There are three home care teams attached to the clinic, each of which is led by a nurse trained in palliative care. Nurses conduct home care visits daily whilst doctors’ visits take place on a weekly basis. Volunteers are responsible for locating the patients in the community. Weekly support meetings for patients with paraplegia and mental illness are held in the clinic.

### Sampling and recruitment

A total of 40 participants took part in the study: eight patients, 14 care-givers, 12 volunteers, three nurses and three doctors. Of these, 25 were female and 15 were male. The background characteristics of the participants are given in Table 1.

**Table 1:** Socio-demographic characteristics of the participants who were interviewed

Participant characteristics	Nurses	Doctors	Volunteers	Patients	Care-givers
Total	3	3	12	8	14
Gender					
Male	0	3	7	3	2
Female	3	0	5	5	12
Age group (in years)					
18–30	1	1	4	2	2
31–50	2	2	7	5	6
51–70	0	0	1	1	6
Number of years in the current role					
0–1	0	0	0	–	6
2–5	1	1	7	–	3
6–10	2	0	2	–	2
11–21	0	2	3	–	3

Each group of research participants – patients, care-givers, volunteers, doctors and nurses – was selected in a different way. Convenience sampling was used to enrol patients for the FGD. Patients who were already familiar with the clinic through attending support group meetings were invited. These included patients with muscular dystrophy (*n* = 3), hemiparesis (*n* = 2), post-polio paralysis (*n* = 1), recurrent hip dislocation (*n* = 1) and psychosis in remission (n = 1). Many of the patients with other conditions were bedridden and were thus not invited as they would not have been able to attend the clinic. Male and female patients were invited to the FGD to ensure that the experiences of both genders were captured.

Purposive sampling was used to recruit care-givers into the study. To ensure maximum variation in the collection of experiences of care-givers, patients with a range of different conditions were identified from clinic records and then their care-givers were contacted. This sample included care-givers of patients with cancer, cardiovascular accidents, mental illness, dementia, chronic diabetes, paraplegia and chronic respiratory disease. The care-givers of male and female patients from each disease category were interviewed at their homes after securing an appointment with them.

As there are only three doctors and three nurses working in the palliative care programme, all were invited for interview. A convenience sample of clinic volunteers was taken based on their availability and interest in participating in the study. Separate FGDs were conducted for male and female volunteers. All doctors, nurses and volunteers were informed about the study during their routine work in the clinic.

### Data collection and study procedures

A total of three FGDs (one with patients and two with volunteers) and 20 in-depth interviews were conducted (Table 2).

**Table 2:** Overview of study participants participating in focus group discussions and in-depth interviews

Participants	FGD (*n* = 3)	IDIs (*n* = 20)
Patients	8 (3 male, 5 female): 1 FGD	0
Care-givers	0	14 (2 male, 12 female)
Doctors	0	3 (3 male)
Nurses	0	3 (3 female)
Volunteers	12 (7 male, 5 female): 2 FGDs	0

*FGD* = Focus Group Discussion, *IDI*=In Depth Interviews

The PI (RRP) is a female medical doctor with training in qualitative research methods. EV, JP and SP are trained researchers. AM and SP have extensive experience in community based palliative care, and have been involved in the programme since its inception in Kerala.

Separate IDI and FGD guides were developed for each group of participants. The guides were piloted in another palliative care unit in the district. FGDs with volunteers and patients and IDIs with doctors and nurses were carried out in the clinic. Interviews with care-givers were carried out in their homes. FGDs lasted an average of 77 min (range: 50–105). IDIs with care-givers lasted an average of 20 min (range: 12–60) and interviews with doctors and nurses an average of 65 min (range: 30–100).

The PI did not have a prior relationship with any of the participants and was not involved in the provision of their medical care. A programme volunteer introduced the PI to the care-givers in their homes, but was not present during the interviews.

The research was explained to participants by the PI, who informed them that this study was taking place as part of an operational research training course and had been developed because of her own interest in the palliative care programme. All participants were informed that it was hoped the study would improve the researchers’ understanding of the programme.

FGDs were audio-recorded and supported by two Malayalam speaking note-takers. All interviews and FGDs were conducted in Malayalam, manually transcribed in Malayalam from the audio-recordings and a selection from each category were translated and typed into English to allow for further review with the other co-investigators [EV & JPT]. Memos were also taken by the PI throughout the data collection period. The FGD and IDIs were conducted between March and June 2017.

### Data analysis

Each Malayalam transcript was read several times by the PI, and with two other co-investigators [AM & SP]. The transcripts were then manually coded, and the codes discussed between the co-investigators to clarify any differences of opinion or misunderstandings. The codes were then organised into categories and common themes between them identified. The themes were then compared across categories to identify and describe the overarching findings of the study [22]. Throughout the analysis process, any discrepancies between the researchers’ interpretation of the data were resolved through discussion and referral back to the original audio files where necessary. The findings have been reported by using Consolidated Criteria for Reporting Qualitative Research guidelines [23].

## Results

A total of 143 individual codes were generated from the transcripts, and similar codes were combined to form a total of 23 broad categories. These categories were compared with each other to explore the overarching experiences of home-based care from the perspective of the different participants. The three main themes which are presented below with examples are: the roles of volunteers, nurses and doctors in the palliative care programme; the benefits and challenges of the programme; patient and care-giver experiences and the role of the community in the provision of palliative care.

### Roles of volunteers, doctors and nurses in the palliative care programme

Doctors, nurses and volunteers described their roles in the programme and discussed how they worked with the other team members to provide medical care to patients at home.

Doctors said that their ‘*first and foremost role’* is to provide clinical care. They determine the management plans for the interim illnesses of patients, communicate prognosis and treatment options by ‘*giving them the facts’* and facilitate referrals and follow up.

The doctors’ home visits were described as limited in frequency compared to those of nurses, and as such they saw themselves as having a coordination role in ‘guiding’ the other team members:

Our main role is that of a team leader. The most important task is to guide other members of the team [in patient care]. Our direct activities [in home care] are fewer, and our indirect activities are more.

40 year old doctor with 14 years’ of experience in palliative care.

Doctors also stated that understanding the non-physical needs of patients, such as their financial problems and psychological and emotional issues, is essential for helping them to plan and manage the patient’s condition. Their role as active listeners gave patients ‘*a chance to vent’*, and psychological, emotional and financial problems (described as psycho-social problems by doctors, nurses and volunteers) were commonly observed.

Doctors and nurses believed that the prolonged time they spend with the patients, in addition to repeated follow up visits, facilitated the development of trust between patients and care providers and enabled patients to share their psychosocial problems.

This doctor summarized the critical role of the doctors in the programme development in the following way:

Doctors had a role in the programme from the beginning. Their vision helped in the development of the programme.

41 year old doctor with 15 years’ of experience.

Nurses enrolled patients into the clinic after a detailed assessment of their medical, social, economic and psychological status. The extent of the involvement of nurses beyond assisting the patients with medical needs was highlighted by this nurse with three years of experience in palliative care, who explained how they ‘*reach as far as the kitchen of the house*.’ Nurses’ tasks included teaching the primary care-givers how to care for bedridden patients so as to prevent pressure sores, and advising care-givers on bowel and bladder care and personal hygiene for the patient.

Volunteers identified a diverse set of roles in the palliative care programme. One volunteer identified their main role as being a ‘*mediator’* between the patients and the community.

The main role of the volunteer who is working as a mediator is to link the isolated patient to the community and provide [them with] more social support. That is, presenting the problems of the isolated patients before the community and informing the related agencies. This is the basic responsibility [of the volunteer].

18 year old male volunteer with 2 years’ experience.

Volunteers also prioritise patients for economic assistance to ensure that the neediest patients receive assistance first.

Volunteers also play a major role in ‘*lighten[ing] the burden of the family’* by carrying out household tasks such as cleaning, feeding and bathing patients, cutting patients’ nails and hair, shaving them and assisting families with applications for financial grants and benefits. They identified their ‘*small, small interventions’* as playing a ‘*big, big role’* in the programme*.*

Volunteers shared the belief that they play a critical role in supporting care-givers who can often experience fatigue after providing long-term care to the patients:

The volunteer is a strong support to the care-giver. Sometimes it is the care-giver who has more psychological issues than the patient!

34 year old male volunteer with 3 years’ experience.

Many volunteers said that they shared a good bond with patients and families because of their frequent home visits, which give patients a chance to share their emotional and psychological problems. Several programme volunteers felt that their role was more important that medical care, and ‘*above that of the doctor’.*

It was evident that the care providers were playing interdependent and complementary roles in the provision of home-based care. Nurses assessed the medical and non-medical needs of patients and discussed them with doctors and volunteers. While doctors guided the medical care of the patient, volunteers addressed the psychological and economic needs of the patient by mobilizing support from local community and neighbourhood networks, in addition to supporting the care-giver psychologically and physically. This interdependent way of working was further facilitated by weekly team meetings in which doctors, nurses and volunteers reviewed the home care visits and discussed the needs of each patient.

### Experiences of care-givers

Interviews with care-givers, who were mostly female, revealed that their tasks were physically demanding and often required them to support their spouses, fathers-in-law or sons with daily activities such as bathing and lifting them out of bed. Financially, many found it difficult to afford daily expenses, including medications, particularly if the person they were caring for was male and had been the main breadwinner for the family. Three categories relating to the experience of caregivers emerged during data analysis, namely the physically demanding, psychologically stressful and socially restrictive nature of their work.

This 37 year old female caring for her mother who had suffered a stroke described it this way:

I have to do everything for my mother. Feed her, wash her after she goes to toilet, bathe her, brush her teeth, comb her hair.

Mothers who care for their sick children reported experiencing stress as a result of the debilitating condition of the children, and worried over their future care. A 34 year old woman caring for her teenage son who was bedridden with cerebral palsy shared that:

My heart aches for my son when I see other children of his age [in the neighbourhood] going to school or when his sibling goes out to play football. This is my greatest agony. It will be there all my life.

This 63 year old male caring for his mother-in-law described his loneliness:

I am all alone here during the day. I have lost touch with many of my friends because I cannot go out leaving her alone.

The psychological stress of care-givers who had been supporting their relatives for many years was also noted by the volunteers and doctors as well.

[They would say] ‘earlier my wife responded whenever I called. Now even if I call five or ten times she doesn’t come.’ They call regularly for necessary and unnecessary things, so naturally the wife may not see it as an urgent need and may not respond. But this will create psychological issues for the patient: earlier it was like this, now nobody is listening.

48 year old volunteer with 14 years of experience

[I]f someone is caring for a sick person they will [be treated with some respect], but for this category [dementia patients] it is the opposite. When they help the patient take a bath, the patient will verbally abuse them. However much they do, they get a directly opposite reaction. Because of this there will be tension between patient and care-giver.

30 year old doctor with 3 years of experience

### Role of the community

The community is directly involved in the programme by ‘*providing cash and kind’*, as one doctor stated.

Volunteers gave examples of instances where organizations in the local community had helped in improving the housing conditions of patients by providing financial support for the plastering of the walls and the construction of bathrooms.

The vital role of economic contributions from the community was described in the following way:

This [the activities of the clinic] is run by the community, not us as volunteers. If the community does not give money for the activities of the clinic, we will cut activities one by one. This is the responsibility of the community.

46 year old male volunteer with 15 years’ experience.

Nurses, doctors and volunteers also believed that the patient’s neighbouring communities had a critical role in supporting patients and families with palliative care needs.

There are neighbours who take care of patients living alone. They cook food and do wound dressings [for the patients].

Palliative care nurse with 3 years’ experience.

This was also suggested by this memo written after visiting an elderly care giver (Table 3).

**Table 3:** Case Based Memo

Memo written after interviewing the care-giver of an elderly male with dementia	
This was quite an eye-opening interview in the sense that I learnt the importance of neighbourhood networks. The caregiver, the wife of a 79 year old man, was very welcoming. The poor condition of the house struck me as I sat down in their one room mud house with unplastered walls. They were very poor with no source of income and they have no children. I learnt the difficulties of being the lone caregiver of a demented patient. Her words “he is just like a child” summarise the amount of patience required to care for him. My question is how does she, an old woman in her early 60s manage it all alone? From her accounts, they receive a complete package of care from the palliative care clinic. But I also learnt about the presence of a very supportive neighbour. I saw the neighbourhood network at play when she recounted how her neighbour helped her to locate her husband when he wandered away from home one morning. She said this neighbor offered his help for any emergency situation she may have. I also got a glimpse of the spectrum of services offered by the palliative care clinic when she told me that volunteers fixed their leaking roof and that one of them sponsors their grocery shopping. The palliative care nurse cuts the patient’s nails, shaves him and cuts his hair. I also learnt that volunteers helped her to take care of her husband when he was hospitalized. I was amazed at her calm and composed attitude; and how she did not appear to be worried or anxious about being alone. Is this because of the security she gets from her neighbourhood and the palliative care support she receives? I definitely learnt that neighbourhood support systems are vital for families like this, so I guess this is the informal neighbourhood network that people talk about. I sense that neighbourhoods are very important in supporting palliative care.	

One of the doctors also cited above described how the clinic facilitated the building of community networks.

We create a neighbourhood network in their [patients’] own neighbourhood to offer support, and create a volunteer team which can understand their needs and act accordingly.

Most of the volunteers in the programme are from the local community. One volunteer stated that:

The patient is not only the responsibility of their houses. He is the responsibility of that community. The community has to intervene for the patient. This duty is done by the palliative care volunteers.

43 year old male volunteer with 3 years’ experience.

This doctor attributed the success of the programme to the involvement of the community.

[The palliative care programme] is getting good support from the community. That is why the programme is still running. [Without it] it won’t function so well.

40 year old doctor with 14 years’ experience

### Benefits of the palliative care programme

This 41 year old doctor stated that the palliative care programme benefited marginalized patients by taking care of all their needs including providing everything from ‘*medicines to rice’.*

This was elaborated on by his colleague, a 30 year old doctor, who stated that the holistic approach of the clinic addressed the non-medical problems of the patient as well as providing regular medical care.

Patients will have lot of issues. They will have financial difficulties or transportation issues to see a doctor. It has been possible [for the palliative care clinic] to give prompt follow up for a good number of patients with chronic illness… it’s not just a medical approach, we provide financial help, social and spiritual support.

Another doctor stated that home care teams reassure families because of their availability and because they enhance ‘*the self-worth’* of patients.

We [the home care team] are able to give regular care. [The patients] get the feeling that whatever is humanly possible is being done for them. Care-givers are well supported and they know they have someone to call when there is a difficulty, someone who can understand their problems. They are getting reassurance.

40 year old doctor with 15 years’ of experience.

Several caregivers, such as the one cited below, attested to these medical benefits and appreciated the convenience of the home care visits conducted by doctors and nurses.

Whenever they [the home care team] come, they check the blood pressure and blood sugar. So, we get these things done without going to hospital. That is a great convenience for us. We can care for him at home.

38 year old female caring for her father-in-law.

Care-givers were also given education and information that could help them to improve the support they gave their relatives so they could adhere to their treatment:

His [father’s] thought was that if the disease subsides after taking medicine then he wouldn’t have to take drugs daily. We had that kind of information. We wouldn’t have done it [stopped taking medication] otherwise. We started taking medicines regularly after coming into the palliative care programme…it’s so much better.

60 year old female care-giver for a male with psychiatric illness.

Care-givers, such as the woman cited above who was caring for her father-in-law also felt empowered to take on basic medical tasks such as wound dressing after receiving training from nurses, which in turn reduced the number of hospital visits they had to make:

They taught us how to do dressings. How to make the water hot for the dressing, to put salt in it, to steam it. We didn’t know any of these things. We don’t see this in hospital [because] they do the dressing for us and we don’t know how it is done.

Patients also appreciated the income-generating activities offered, including making soap, soap powder and umbrellas. In addition, they welcomed the peer support network and independence the programme provided:

I felt that I could do more after [receiving] palliative care. Now I am mentally and physically strong. I got financial support also. I started earning after learning soap and umbrella making. I am self-reliant now. It was possible because of the strong commitment and support of each and every volunteer.

36 year old male patient with Muscular dystrophy.

Patients reported that they grew in confidence from attending the support group, which helped them become more independent in their everyday lives:

I became brave after attending the support group. [I can] go everywhere. Earlier if I was asked to go somewhere I would not go. I needed someone to go with me. Now I don’t need anyone. I am ready to go anywhere [in my wheelchair].

29 year old female patient with Muscular dystrophy.

The programme gave some patients a sense of activism, and they campaigned in their communities on issues including wheelchair access and community attitudes towards disability.

This female volunteer with over 20 years’ experience in the programme shared how she believed palliative care had made a difference to patients over time.

In the earlier days wherever there were wounds, there were maggots. Today we still see wounds but there are no maggots. That is because of palliative care. We [volunteers] have crossed streams and rivers to ferry paraplegia patients; often three or more [volunteers] lifting one patient. When we held their hands, the world saw them. They [paraplegia patients] saw the world outside. That’s the change in their lives. It’s just because of palliative care. Otherwise they would still be living like cavemen…

### Challenges of the programme

Care providers admitted that they were not able to reach all the patients in the municipality who needed palliative care. This was due to a combination of factors related to providers and patients. The provider related factors included a shortage of staff to cope with increasing patient numbers, as this 30 year old doctor stated:

The number of patients is rapidly increasing. We are finding it difficult to find and reach all patients [in the county]. Despite seven days of home care visits we are unable to reach everywhere.

The difficulty in recruiting doctors, nurses and volunteers for home-based care was highlighted by this volunteer:

We have a shortage of trained nurses and a high shortage of trained volunteers. Similarly, doctors. Even today’s newspaper has an advertisement seeking doctors for palliative care!

46 year old male volunteer with 15 years’ experience.

Patient related factors which prevented families from seeking palliative care were linked to the perceptions some people had about the programme, as the doctor cited above explained:

Some people think that palliative care is a service only for the poor. Actually, palliative care is for all patients. The effect [of disease] is the same for rich and poor.

Doctors and nurses shared other perceptions of patients and families which made them reluctant to seek palliative care; including the open nature of the programme which made it difficult for patients to keep their disease private. One of the nurses shared that palliative care instils a fear of death in some patients and families as they believe that palliative care is for the dying.

## Discussion

This study describing a twenty-year-old home-based palliative care programme showed that, despite challenges, the programme was able to provide patients and their families with essential, long-term support. The programme was perceived to improve the lives of people with longstanding conditions and terminal illness through the provision of medical and psycho-social support to patients and their families. Community volunteerism was also a key element of the programme.

The roles of the volunteers, doctors and nurses in the provision of palliative care are interdependent and complement each other to ensure the basic needs of the patient are met [24]. The need for home-based palliative care teams to function as a single unit in the best interests of the patients is shared by professional palliative care providers from other contexts, including Ontario and Norway [25, 26]. The close collaboration and communication between patient, family and home care team is reported to optimize home based palliative care.

It was interesting to note that everyone in the current programme thought that their role was essential, and often the most important, to the provision of home-based care. Doctors saw themselves as the leaders of the programme, the volunteers saw themselves as having a higher status than doctors, and the nurses claimed that their role was most critical.

### Provision of holistic care

The primary focus of home-based palliative care has typically been symptom management, and in many contexts, there is often a focus on end of life care [24]. However, some studies have reported that patients receiving palliative care require help with social issues as well [24, 27]. Although strong palliative care teams are those that deliver timely and accessible medical care, palliative care provision often goes beyond clinical tasks [24, 25]. Most of the patients and care-givers interviewed faced socio-economic problems caused by loss of wages and treatment expenditure. The care providers believed that these psycho-social issues also needed support and intervention to improve the quality of life of patients. Communication skills were considered vital for this process. A similar perspective is shared by care-givers from other palliative care settings [9, 24, 25].

- The current programme has been beneficial in making medical care regularly available to patients who are marginalized from regular health care. In resource poor settings, health care is costly due to both direct and indirect costs. The cost-effectiveness and convenience of home-based care programmes are well documented elsewhere [28–30]. Care-givers reported that the home care visits of professionals reduced hospital visits and admissions. Home visits in other contexts have also been reported to reduce admissions to emergency departments, which are often stressful and exhaustive for patients and their families [31]. A meta-ethnographic study has shown that the availability and skill of home care teams can also enhance the patient’s feelings of security at home [9].
- There is often a preconceived idea that palliative care is directly linked to active dying, and that supposed link can generate fear in some patients and their families [8]. This was reported as a barrier in accessing palliative care in our study, thus the importance of educating patients about the benefits of palliative care may alleviate fears and encourage them to access services in this context as well as elsewhere [8].

### Family care-givers

Studies report that allowing care-givers to assist with medical treatment and physical care is beneficial as it means the patients’ daily life is less dependent on the schedule of professionals, in addition to making it cost-effective [32].

The role of the care-giver - usually a female family member - is very important to consider as they are the ones who provide daily care to bedridden patients. They bear the burden of physical and psychological exhaustion in addition to experiencing social isolation as a result of the demands of caring for someone else. The gendered dimension in care-giving raises issues of social justice, gender and power dynamics in the experience of providing palliative care [33].

### Role of the community

A greater sustainability of resources is required to achieve good quality palliative care, which Kerala has achieved through creating and supporting community networks that respond to the needs of marginalized patients and their families [34]. The programme is distinct in its ownership by the local community and is sustained by volunteerism and the financial, material and human resource contributions of local individuals. The programme has been running in this mode for twenty years, showing the motivation of the local community to sustain it. Volunteers believe that the visible benefits to patients in turn enhance the trust of the community in the programme, which further motivates it to sustain and support it. Maintaining this degree of volunteerism remains, however, a challenge.

### Strengths

One of the main strengths of this study is the richness of the data, and the similarities in themes identified during FGDs and interviews with different groups of participants. This is the first qualitative study conducted with this particular population in Kerala and offers a unique insight into the experiences of those living with and those caring for terminal and chronic illnesses.

### Limitations

It was not possible to include patients from all categories of diseases/conditions who are supported by the programme, as many of them were too sick, bedridden or unable to talk. Health-care workers may have been reluctant to criticise the programme as it might have an impact on their jobs, thus creating a degree of bias in their responses. Not all transcripts were translated into English, meaning that some detail may have been lost as only two co-investigators were able to review all the original language transcripts.

## Conclusion

The results from from this study show the importance of palliative care in this community in Kerala from the perspective of those providing and receiving services. The programme is sustained by the volunteerism of the local community. The salient features of this programme are the availability of regular holistic medical care, the empowerment of family care-givers and the provision of psycho-social support. This home-based palliative care programme was perceived to improve the lives of patients and their care-givers despite the challenges of maintaining a programme which was volunteer-led.
